# Therapeutic outcomes of cryotherapy plus Graston Technique on pain and activities of daily living in individuals with knee pain

**DOI:** 10.3389/fmed.2026.1836112

**Published:** 2026-06-17

**Authors:** Vanitha Innocent Rani, Akshaya Saikannan, Manal Ibrahim Alrefaee Asiri, Abhilash Venunathan, Kandasamy Muthugounder, Montaha Mohamed Ibrahim Mohamed, Shobana Gangadharan, Amna Hussian Madani Elfeel, Fatima Adam Gaber Mustafa, Jayaprakash Kanthasamy, Francis Moses Rajappa, Mahendran Kaliyamoorthy

**Affiliations:** 1 Department of Community and Mental Health Nursing, King Khalid University, Mahayil, Asir, Saudi Arabia; 2 Department of Anatomy, Vinayaka Mission’s Medical College and Hospital, Vinayaka Mission Research Foundation (DU), Karaikal, India; 3 Department of Community Health Nursing, Nursing College, King Khalid University, Asir, Saudi Arabia; 4 Department of Medical Surgical Nursing, Co-operative College of Nursing Payyanur, Kerala University of Health Sciences, Kerala, India; 5 Department of Community and Mental Health Nursing, College of Nursing, Khamis Mushait, King Khalid University, Abha, Saudi Arabia; 6 Department of Adult and Advanced Nursing Care, King Khalid University, Nursing College, Mahayil, Asir, Saudi Arabia; 7 College of Health Sciences, University of Buraimi, Al-Buraimi Governorate, Oman; 8 Department of Medical and Surgical, Nursing College, King Khalid University, Mahayil, Asir, Saudi Arabia; 9 Department of Maternity and Child Health Nursing, Nursing college, King Khalid University, Mahayil, Asir, Saudi Arabia; 10 Department of Addiction, GRN, Naufar Center under the Ministry of Public Health, Doha, Qatar; 11 St. Xavier College of Nursing, Kumbakonam, Tamil Nadu, India; 12 ViMah’s Sports and Orthopedic Physical Therapy Centre, Tamil Nadu, Thanjavur, India

**Keywords:** ADL, cryotherapy, Graston Technique, instrument-assisted soft tissue mobilization, knee pain, rehabilitation, pain intensity

## Abstract

**Aims:**

This study evaluated the effectiveness of combining cryotherapy with the Graston Technique in reducing pain and improving activities of daily living (ADL) among individuals with non-traumatic knee pain.

**Methods:**

A total of 100 participants aged 31–50 years were recruited from August to October 2024. Participants were randomly assigned to either an experimental group (Graston Technique + cryotherapy) or a control group (Graston Technique alone), with 50 participants in each group. Both groups received one treatment session daily for 10 consecutive sessions over 2 weeks, with each session lasting 30–35 min. The Graston Technique was applied using standardized stainless-steel instruments over the quadriceps, hamstring, and peri-patellar regions. In the experimental group, cryotherapy was applied immediately after treatment using cold packs maintained at 10 °C–12 °C for 10–15 min. Pain intensity was assessed using the Visual Analogue Scale (VAS), while functional performance was evaluated using ADL tasks including walking, stair climbing, cross-leg sitting, and toilet activities. Data were analyzed using paired and independent *t*-tests, with statistical significance set at *p* < 0.05.

**Results:**

Both groups demonstrated significant improvements following intervention (*p* < 0.001). The experimental group showed greater pain reduction (mean difference: 2.18 ± 0.48) and better improvement in ADL performance (mean difference range: 2.16–3.12) compared with the control group. The control group showed moderate improvements in pain (0.56 ± 0.50) and ADL scores (1.32–1.62). Correlation analysis revealed a strong positive association between pain reduction and improved ADL performance (*r* = 0.71, *p* < 0.001).

**Conclusion:**

The findings suggest that combining cryotherapy with the Graston Technique is more effective than the Graston Technique alone in improving pain and functional ability among individuals with non-traumatic knee pain. This combined intervention may serve as a practical, non-invasive rehabilitation strategy in physiotherapy settings. Future studies should include larger sample sizes and longer follow-up periods to evaluate long-term clinical outcomes.

## Highlights

- Combined cryotherapy + Graston therapy produced greater knee pain reduction.
- ADL functions improved significantly in the experimental group.
- VAS scores showed larger reductions with the integrated intervention.
- Functional mobility gains exceeded those of the control group.
- Multimodal therapy offers an effective, non-pharmacologic knee pain solution.

## 1 Introduction

Knee joint pain is a prevalent musculoskeletal complaint affecting adults worldwide, often resulting from degenerative, inflammatory, or traumatic conditions that impair daily living and mobility (1). It substantially limits activities such as walking, stair climbing, and sitting, thereby reducing quality of life and work productivity (2). Importantly, knee pain is strongly associated with limitations in activities of daily living (ADL), including walking, stair climbing, and transitioning from sitting to standing, highlighting the need for interventions that improve both pain and functional outcomes (3, 4). Conventional management approaches for knee pain include pharmacologic therapy, exercise, cryotherapy, and manual or instrument-assisted soft-tissue techniques (IASTM), which aim to relieve pain and improve range of motion (ROM) (5).

Cryotherapy, or localized cold therapy, is widely used for its analgesic and anti-inflammatory effects in musculoskeletal rehabilitation. By reducing tissue temperature, it lowers metabolic demand and nerve conduction velocity, thereby diminishing inflammation, swelling, and pain perception (6, 7). Different cryotherapy approaches, including localized cold packs, cold-water immersion, and whole-body cryotherapy demonstrated beneficial effects on soft tissue recovery and joint-related conditions, although treatment efficacy may vary depending on application duration and frequency (8–10).

The Graston Technique®, an IASTM method, employs specially designed stainless-steel instruments to detect and treat areas of soft-tissue fibrosis, chronic inflammation, and fascial restrictions (5, 11, 12). By applying controlled microtrauma to the affected area, the technique promotes collagen realignment, tissue remodeling, and restoration of functional movement (5, 11). Previous studies have shown that IASTM can effectively reduce pain and improve ROM in individuals with tendinopathies, myofascial adhesions, and other musculoskeletal disorders (13). Consequently, the Graston Technique has gained increasing attention in physiotherapy and sports rehabilitation settings as a non-invasive intervention for soft tissue dysfunction (12, 14).

Prior studies have highlighted the effectiveness of advanced cooling protocols in reducing pain and swelling following sports injuries and postoperative rehabilitation, with emerging evidence also supporting their use in non-traumatic knee pain (15–20) Additionally, several functional assessment tools have been developed to evaluate knee-related disability and rehabilitation outcomes (21). Most previous studies have evaluated cryotherapy (9, 22, 23) or IASTM as separate interventions (24, 25), while limited evidence exists regarding their combined therapeutic effects on pain and functional recovery. Recent fascia-oriented rehabilitation approaches and IASTM, have also shown benefits in improving mobility, tissue flexibility, circulation, and functional recovery while reducing pain and muscle stiffness (26). Despite these advances, important research gaps remain.

Furthermore, although cryotherapy is effective for short-term pain relief, its influence on ADL-related functional performance has not been sufficiently explored (27). The potential role of cryotherapy in enhancing manual therapy outcomes by reducing post-treatment soreness and improving tissue extensibility also remains under-investigated.

Additionally, the potential for cryotherapy to enhance manual therapy outcomes by reducing post-mobilization soreness and improving tissue pliability remains understudied. While both cryotherapy and Graston Technique have demonstrated individual benefits, few studies have examined their combined therapeutic impact on knee pain and functional mobility (28). Integrating cryotherapy with IASTM may enhance recovery by simultaneously modulating inflammation and facilitating tissue repair. From a rehabilitation perspective, combining cryotherapy with the Graston Technique may provide synergistic therapeutic benefits by simultaneously reducing inflammation and promoting soft tissue recovery. However, few studies have examined the concurrent use of these interventions in individuals with non-traumatic knee pain, and even fewer have focused on clinically meaningful functional outcomes such as ADL performance (13, 14, 29).

Thus, the present study was designed to address this research gap by evaluating the combined effects of cryotherapy and the Graston Technique on pain reduction and functional improvement among adults with non-traumatic knee pain. Emerging evidence from fascia-oriented rehabilitation approaches, including foam rolling and tissue-focused interventions, has demonstrated improvements in tissue flexibility, circulation, mobility, and functional recovery (26). Based on previous findings supporting the individual benefits of cryotherapy and IASTM, it was hypothesized that the combined intervention would produce greater reductions in pain and improved ADL performance compared with the Graston Technique alone.

## 2 Materials and methods

### 2.1 Study design and setting

An experimental assessor-blinded study design was conducted at the Vimha Rehabilitation Center, Tanjore, India, between August and October 2024. The study compared the effects of the Graston Technique combined with cryotherapy (experimental group) and the Graston Technique alone (control group) on pain and functional outcomes among individuals with non-traumatic knee pain.

To minimize potential expectation bias, both groups received treatment under similar clinical conditions, including equal session duration, therapist attention, treatment frequency, and rehabilitation settings. Participants were randomly assigned into two groups using a simple lottery method. Pre- and post-treatment assessments were performed by trained physiotherapists who were blinded to group allocation. Participant blinding was not feasible because the cold sensation associated with cryotherapy could be easily recognized. However, participants were not informed regarding the expected superiority of either intervention, thereby helping to reduce placebo-related influences.

Ethical approval was obtained from the Institutional Ethics Committee (IEC/VIMAH/#2024-010), and all participants provided written informed consent prior to participation. The study adhered to the ethical standards of the Declaration of Helsinki.

### 2.2 Participants and sample

Participants were recruited from the Vimha Rehabilitation Center, Thanjavur, India, during the study period. Individuals presenting with non-traumatic knee pain were screened for eligibility according to predefined inclusion and exclusion criteria. Knee pain was clinically defined based on the presence of persistent musculoskeletal knee pain associated with mild-to-moderate functional limitation during daily activities. The recruited participants primarily presented with early degenerative knee conditions, overuse-related musculoskeletal pain, soft-tissue tightness, patellofemoral discomfort, and non-specific mechanical knee pain without major structural injury.

Eligible participants included adults aged 31–50 years who experienced mild-to-moderate knee pain for more than 2 weeks and were able to perform daily activities independently without assistive devices. Participants with acute traumatic injuries, ligament tears, fractures, recent knee surgery, systemic inflammatory disorders, neurological conditions, recent corticosteroid injections, or contraindications to cryotherapy or manual therapy were excluded. Acute injuries were specifically excluded to reduce clinical heterogeneity and avoid confounding inflammatory responses that may independently influence pain severity, tissue healing, and rehabilitation outcomes.

A convenience sampling method was used to recruit eligible participants. Those who met the study criteria were randomly allocated into two equal groups using a simple random lottery technique. The experimental group (*n* = 50) received combined cryotherapy and Graston Technique treatment, whereas the control group (*n* = 50) received the Graston Technique alone.

The sample size of 100 participants was determined based on previous musculoskeletal rehabilitation studies (30, 31), which demonstrated that a minimum of 40–50 participants per group provides adequate statistical power (80%) to detect significant differences at an alpha level of 0.05.

### 2.3 Intervention

**Figure 1:**
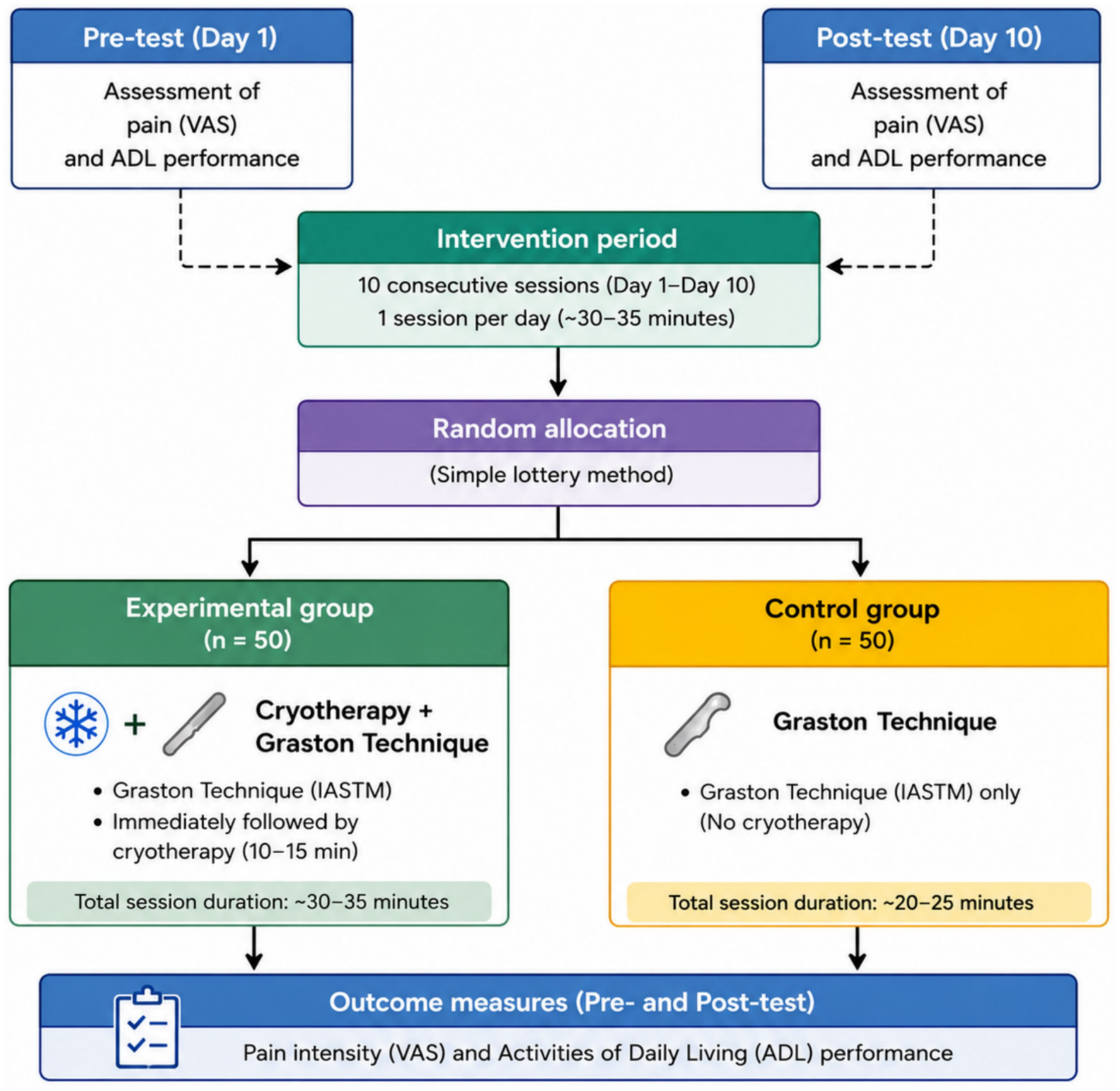
Study design for intervention. VAS, Visual Analogue Scale; ADL, Activities of daily living; IASTM, Instrument-assisted soft tissue mobilization.

Both groups received treatment once daily for 10 consecutive sessions over a 2-week rehabilitation period, administered by licensed physiotherapists trained in the Graston Technique and cryotherapy application (Figure 1). The intervention frequency and duration were selected based on previous musculoskeletal rehabilitation studies demonstrating the safety and effectiveness of short-term repeated IASTM and cryotherapy protocols for pain reduction and functional recovery (11, 28, 31). To minimize the risk of tissue irritation and ensure patient safety, moderate treatment intensity was consistently maintained throughout the intervention period, and all procedures were performed under standardized clinical supervision at the Vimha Rehabilitation Center, Thanjavur, India. Participants were monitored daily for discomfort, excessive soreness, or adverse reactions, and no treatment-related complications were observed. Each session lasted approximately 30–35 min.

#### 2.3.1 Experimental group (Graston Technique + cryotherapy)

Participants in the experimental group underwent IASTM using the Graston Technique followed immediately by localized cryo-therapy. The Graston Technique was performed using stainless steel instruments of var-ying convex–concave edges to treat soft-tissue adhesions and fascial restrictions. Each session involved five keystrokes sweeping, fanning, brushing, strumming, and scooping applied over the quadriceps, hamstring, and peri-patellar regions. Moderate pressure was maintained to elicit mild erythema, indicating increased microcirculation. Following IASTM, cryotherapy was applied using a cold pack maintained at 10 °C–12 °C for 10–15 min to the treated area. The application area was covered with a thin towel to prevent frostbite. Cryotherapy was administered to reduce post-treatment soreness, inflammation, and localized pain. The combined session was designed to promote tissue recovery, decrease neural sensitivity, and improve overall mobility and comfort.

#### 2.3.2 Control group (Graston Technique only)

Participants in the control group received only the Graston Technique intervention using the same treatment procedures, anatomical regions, instruments, session frequency, and therapist instructions as the experimental group. Each session lasted approximately 20–25 min, and no cryotherapy was administered following treatment. This design allowed direct evaluation of the additional therapeutic effect of cryotherapy beyond standard IASTM treatment.

To avoid potential confounding effects, participants in both groups were advised not to undergo additional physiotherapy treatments, structured exercise programs, or pain-related interventions during the study period. Compliance was monitored daily throughout the intervention period.

### 2.4 Measures

#### 2.4.1 Pain intensity (VAS)

Pain intensity was assessed using the Visual Analogue Scale (VAS), a 10-cm horizontal line anchored by “no pain” (0 mm) on the left and “worst imaginable pain” (100 mm) on the right (32). VAS is a widely adopted instrument for assessing pain intensity with greater precision than categorical measures. It consists of a 10 cm line either horizontal or vertical anchored by “no pain” at one end and “worst imaginable pain” at the other. Participants indicate their pain level by marking a point along the line, and the distance from the “no pain” anchor is measured to generate a score ranging from 0 to 100. VAS is valued for its high sensitivity in detecting subtle changes in pain levels and has demonstrated strong reliability, with test–retest coefficients frequently exceeding 0.90. However, its accuracy depends on the participant’s ability to understand the scale, making it less suitable for individuals with cognitive limitations.

#### 2.4.2 Functional performance (ADL)

Functional ability was evaluated using ADL assessments, focusing on four key functional domains: walking, cross-leg sitting, stair climbing, and toilet activities (33). Each activity was rated on a five-point Likert scale ranging from 0 = no difficulty to 4 = extreme difficulty, reflecting the participant’s level of functional limitations. Higher scores indicated greater impairment in daily performance. The ADL measure provides a simple, valid indicator of mobility and independence, allowing quantitative comparison of pre- and post-intervention functional changes.

### 2.5 Data collection

Data collection for this experimental study was conducted from August to October 2024 at Vimha Rehabilitation Center, Thanjavur, India. Participants aged 31–50 years with clinically diagnosed knee joint pain were screened for eligibility according to the inclusion and exclusion criteria. Eligible participants were divided into two groups: the experimental group (Graston Technique combined with cryotherapy) and the control group (Graston Technique alone), each consisting of 50 participants selected through a random sampling method. Prior to data collection, all participants were briefed about the study objectives and procedures through a detailed information sheet and verbal ex-planation. Written informed consent was obtained in accordance with the Declaration of Helsinki (42). Participation was voluntary, and confidentiality of participant information was strictly maintained throughout the study.

Data was collected at two points: Day 1 (Pre-test) before the initiation of treatment and Day 10 (Post-test) following the intervention period. Pain intensity was assessed using the Visual Analogue Scale (VAS), and functional performance was evaluated using ADL assessments, which included walking, cross-leg sitting, stair climbing, and toilet activities. Trained physiotherapists who were blinded to group allocation performed all evaluations to minimize observer bias.

### 2.6 Data analysis

Data was analyzed using the Statistical Package for the Social Sciences (SPSS, version 26.0; IBM Corp., Armonk, NY). All data were screened for completeness, normality, and outliers. Descriptive statistics (mean, standard deviation, and frequency) summarized participants’ demographic and baseline characteristics. Paired *t*-tests examined with-in-group pre- and post-test changes in pain (VAS) and functional ability (ADL) scores, while independent *t*-tests compared post-test mean differences between the experimental and control groups to assess the additional effect of cryotherapy. Pearson’s correlation analysis evaluated the relationship between pain reduction and functional improvement. One-way ANOVA tested associations between demographic variables and post-test pain scores, and independent *t*-tests were applied for dichotomous variables such as gender and employment. Effect sizes (Cohen’s *d* and η^2^) were calculated to interpret the magnitude of differences. All tests were two-tailed, with *p* < 0.05 considered statistically significant.

## 3 Results

### 3.1 General characteristics of the study participants in experimental and control groups

Table 1 presents the general characteristics of participants in the experimental and control groups (*n* = 100). Both groups consisted of 50 participants each, with a nearly equal gender distribution males accounted for 50% in the experimental group and 48% in the control group, while females represented 50 and 52%, respectively. The majority of participants were in the 46–50-year age range, comprising 30% of the experimental group and 44% of the control group, followed by those aged 31–35 years (42 and 22%, respectively). Most participants were currently employed (66% in the experimental group and 64% in the control group). Regarding body weight, a larger proportion of the experimental group (68%) and the control group (86%) weighed more than 60 kg. The prevalence of chronic diseases was relatively low, with 28% of the experimental group and 22% of the control group reporting at least one chronic condition.

**Table 1:** General characteristics of experimental and control group participants (*n* = 100).

Characteristics		Experimental group (*n* = 50)		Control group (*n* = 50)	
		*N*	%	*N*	%
Age group (years)	31–35	21	42	11	22
	36–40	6	12	11	22
	41–45	8	16	6	12
	46–50	15	30	22	44
Gender	Male	25	50	24	48
	Female	25	50	26	52
Currently employed	Yes	33	66	32	64
	No	17	34	18	36
Weight	<60 kg	16	32	7	14
	>60 kg	34	68	43	86
Chronic disease	Yes	14	28	11	22
	No	36	72	39	78

### 3.2 Comparison of VAS score differences before and after intervention

Table 2 shows the comparison of mean Visual Analogue Scale (VAS) scores before and after the intervention among the experimental and control groups. In the experimental group, the mean VAS score significantly decreased from 3.72 ± 0.45 before treatment to 1.54 ± 0.54 after treatment, yielding a mean difference of 2.18 (*p* < 0.001). In the control group, the mean VAS score also showed a reduction from 4.00 ± 0.42 to 3.44 ± 0.50, with a smaller mean difference of 0.56 (*p* < 0.001). from 4.00 ± 0.42 to 3.44 ± 0.50, with a smaller mean difference of 0.56 (*p* < 0.001).

**Table 2:** Comparison of pre- and post-intervention Visual Analogue Scale (VAS) scores between experimental and control groups.

Group	VAS score	Mean	SD	Mean difference (pre-post)	*p*
Experimental	Pre	3.72	0.45	2.18	<0.001
	Post	1.54	0.54		
Control	Pre	4.00	0.42	0.56	<0.001
	Post	3.44	0.50		

SD, Standard deviation.

### 3.3 Comparison of ADL performance before and after intervention

After the 10-day intervention, both groups showed improvements in ADL, but the experimental group that received cryotherapy combined with the Graston Technique demonstrated a more pronounced reduction in difficulty across all activities than the control group. As shown in Table 3, walking scores improved from 4.24 ± 0.77 to 1.12 ± 0.33 (mean difference = 3.12) in the experimental group, whereas the control group improved from 5.00 ± 0.00 to 3.38 ± 0.49 (mean difference = 1.62). Similar trends were observed for cross-leg sitting, stair climbing, and toilet activities, where mean improvements in the experimental group ranged from 2.16 to 2.18, compared with 1.32–1.36 in the control group.

**Table 3:** Comparison between mean differences between pre and post between intervention among experimental and control groups.

Group	ADL activity	Pre (M ± SD)	Post (M ± SD)	Mean difference	% Improvement	*p*
Experimental	Walking	4.24 ± 0.77	1.12 ± 0.33	3.12	73.6%	<0.001
	Cross-leg sitting	4.30 ± 0.76	2.12 ± 0.33	2.18	50.7%	<0.001
	Stair climbing	4.30 ± 0.76	2.14 ± 0.35	2.16	50.2%	<0.001
	Toilet activities	4.32 ± 0.77	2.14 ± 0.35	2.18	50.5%	<0.001
Control	Walking	5.00 ± 0.00	3.38 ± 0.49	1.62	32.4%	<0.001
	Cross-leg sitting	5.00 ± 0.00	3.64 ± 0.56	1.36	27.2%	<0.001
	Stair climbing	5.00 ± 0.00	3.68 ± 0.62	1.32	26.4%	<0.001
	Toilet activities	5.00 ± 0.00	3.66 ± 0.59	1.34	26.8%	<0.001

M, Mean; SD, Std. deviation.

### 3.4 Paired *t*-test comparison of pre- and post-intervention scores in an experimental group

**Figure 2:**
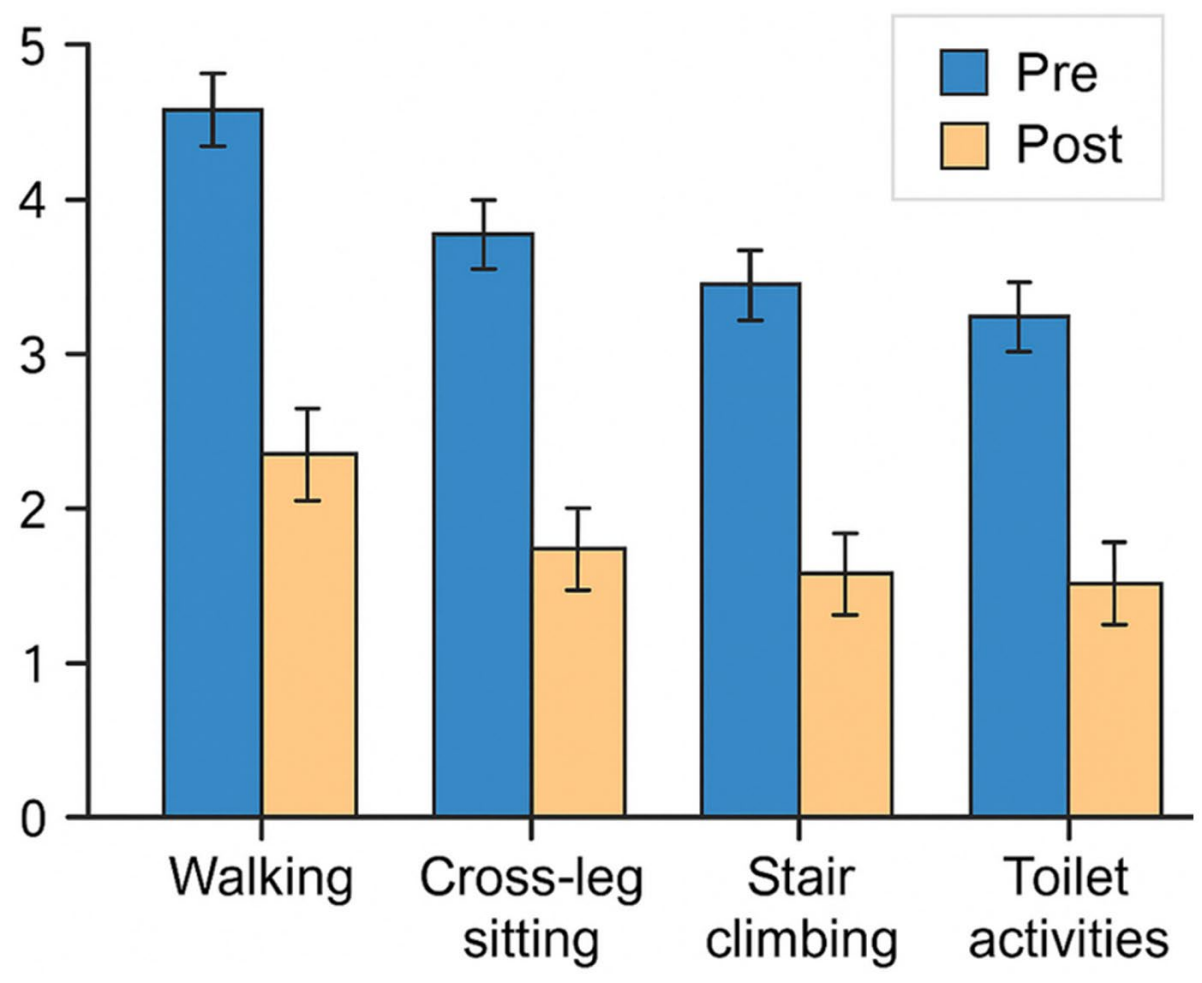
ADL activity comparison of pre and post difference in an experiment group.

Table 4 presents the paired *t*-test analysis comparing pre- and post-intervention outcomes within the experimental group for pain (VAS) and ADL. The findings showed a significant reduction in pain following the combined cryotherapy and Graston Technique intervention, with the mean VAS score decreasing by 2.18 ± 0.48 (95% CI: 2.04–2.32; *t*(49) = 31.99, *p* < 0.001). Significant improvements were also observed across all ADL components. Walking performance improved by 3.12 ± 0.75 (95% CI: 2.91–3.33; *t*(49) = 29.57, *p* < 0.001), while cross-leg sitting improved by 2.18 ± 0.75 (95% CI: 1.97–2.39; *t*(49) = 20.62, *p* < 0.001). Stair climbing showed an improvement of 2.16 ± 0.74 (95% CI: 1.95–2.37; *t*(49) = 20.68, *p* < 0.001), and toilet activities improved by 2.18 ± 0.72 (95% CI: 1.98–2.39; *t*(49) = 21.42, *p* < 0.001) (Figure 2).

**Table 4:** Paired *t*-test comparison of mean differences of within pre and post intervention of experimental groups on VAS and ADL.

Paired test (pre vs post)		Paired differences				*t*	df	*p*
		Mean ± SD	SEM	95% CI				
VAS	Pain score	2.18 ± 0.48	0.07	2.04	2.32	31.99	49	<0.001
ADL	Walking	3.12 ± 0.75	0.11	2.91	3.33	29.57	49	<0.001
	Cross leg sitting	2.18 ± 0.75	0.11	1.97	2.39	20.62	49	<0.001
	Stair climbing	2.16 ± 0.74	0.10	1.95	2.37	20.68	49	<0.001
	Toilet activities	2.18 ± 0.72	0.10	1.98	2.39	21.42	49	<0.001

SD, Standard deviation; SEM, Standard Error Mean; CI, confidence interval; ADL, Activities of daily living; VAS, Visual Analogue Scale.

### 3.5 Paired *t*-test comparison of pre- and post-intervention scores in control groups

Table 5 summarizes the paired *t*-test results comparing pre- and post-intervention outcomes within the control group, which received the Graston Technique alone. The analysis revealed statistically significant improvements in both pain (VAS) and ADL performance after the intervention. The mean VAS score decreased by 0.56 ± 0.50 (95% CI [0.42, 0.70]), *t*(49) = 7.90, *p* < 0.001, indicating a modest but significant reduction in pain intensity. In terms of functional ability, all ADL components also showed significant improvement. Walking improved by 1.62 ± 0.49 (95% CI [1.48, 1.76]), *t*(49) = 23.36, *p* < 0.001; cross-leg sitting by 1.36 ± 0.56 (95% CI [1.20, 1.52]), *t*(49) = 17.09, *p* < 0.001; stair climbing by 1.32 ± 0.62 (95% CI [1.14, 1.50]), *t*(49) = 15.04, *p* < 0.001; and toilet activities by 1.34 ± 0.59 (95% CI [1.17, 1.51]), *t*(49) = 15.98, *p* < 0.001.

**Table 5:** Paired *t*-test comparison of mean differences of within pre and post intervention of Control groups on VAS and ADL.

Paired test (pre vs. post)		Paired differences				*t*	df	*p*
		Mean ± SD	SEM	95% CI				
				Lower	Upper			
VAS	Pain score	0.56 ± 0.50	0.07	0.417	0.70	7.89	49	<0.001
ADL	Walking	1.62 ± 0.49	0.07	1.48	1.76	23.36	49	<0.001
	Cross leg sitting	1.36 ± 0.56	0.08	1.20	1.52	17.09	49	<0.001
	Stair climbing	1.32 ± 0.62	0.09	1.14	1.49	15.04	49	<0.001
	Toilet activities	1.34 ± 0.59	0.08	1.17	1.51	15.98	49	<0.001

SD, Std. deviation; SEM, Standard Error Mean; CI, confidence interval.

## 4 Discussion

This study evaluated the combined effect of cryotherapy and the Graston Technique on pain reduction and improvement in ADL among adults with knee joint pain. The findings demonstrate that participants who received the combined intervention achieved greater pain relief and functional recovery than those treated with the Graston Technique alone. These results suggest that integrating cryotherapy with instrument-assisted soft tissue mobilization (IASTM) may provide additional therapeutic benefits in short-term musculoskeletal rehabilitation.

The primary outcome of this study was pain reduction measured using the VAS. Participants in the experimental group demonstrated a substantially greater reduction in pain scores compared with the control group. This finding is consistent with previous research indicating that cryotherapy reduces pain through several physiological mechanisms, including decreased nerve conduction velocity, reduced metabolic activity, vasoconstriction, and suppression of inflammatory responses (6, 7, 22). Lower tissue temperature may also reduce muscle spasm and neural sensitivity, thereby improving pain tolerance and comfort during movement (34, 35). In the present study, the application of cryotherapy immediately after the Graston Technique may have reduced post-treatment soreness and secondary tissue irritation, contributing to the greater pain relief observed in the experimental group.

Functional improvement in ADL performance was another important outcome of this study. Participants receiving combined cryotherapy and Graston intervention showed greater improvement in walking, stair climbing, cross-leg sitting, and toilet activities than those in the control group. Functional limitations in knee pain are often associated with reduced mobility, soft tissue stiffness, pain-related movement restriction, and impaired joint mechanics [2, 18, 21]These findings are consistent with the established role of cryotherapy in reducing inflammation, muscle spasm, and neural transmission speed, thereby contributing to an-algesia and early recovery (7, 22). Cryotherapy also promotes vasoconstriction and limits secondary tissue damage, which is crucial in sub-acute musculoskeletal conditions (10).

The present findings align with earlier studies that have reported significant reductions in pain and improvement in soft tissue flexibility with cryotherapy combined with manual therapy. Prior studies found that localized cold application immediately after exercise reduced post-exercise soreness and improved joint mobility (6, 9, 10, 22, 34, 35). Similarly, study reported that cryotherapy decreased muscle temperature and improved tolerance to stretch, facilitating more effective manual therapy outcomes (27, 36, 37). These physiological mechanisms support the observed improvements in pain and ADL scores in this study.

In parallel, the use of the Graston Technique has been well documented for its efficacy in managing soft tissue dysfunction. Studies demonstrated that IASTM promotes collagen remodeling, increases tissue extensibility, and enhances circulation, resulting in improved range of motion and pain reduction (5, 25). Previous studies have independently demonstrated the benefits of cryotherapy and IASTM in musculoskeletal rehabilitation (8, 10, 15, 27). Author further suggested that IASTM facilitates fibroblast proliferation and angiogenesis, accelerating healing in chronic musculoskeletal injuries (38, 39). The present results agree with these findings, as participants treated with the Graston Technique combined with cryotherapy achieved superior improvements in both pain and functional performance.

The present study also demonstrated that treatment effects were relatively consistent across demographic characteristics, including age, gender, employment status, and body weight. This finding indicates that the combined intervention may be broadly applicable across different patient subgroups with non-traumatic knee pain. Similar observations have been reported in previous physiotherapy-based rehabilitation studies, where treatment outcomes were more strongly associated with intervention quality and consistency than demographic variation (2, 40). Within-group analyses confirmed significant pre- to post-intervention gains in both pain relief and ADL scores for the experimental group, underscoring the combined treatment’s clinical value (19). Although the control group receiving Graston therapy alone also improved, the magnitude of change was notably smaller. These results support prior evidence that multimodal interventions integrating manual therapy with adjunct modalities yield greater rehabilitation benefits than single-technique approaches (31). Cryotherapy likely enhanced the tissue recovery process initiated by IASTM by reducing post-treatment soreness and facilitating muscular relaxation, thereby improving participants’ ability to perform daily tasks such as walking, stair climbing, and sitting.

Regression analysis revealed no significant association between demographic characteristics (age, gender, employment status, weight, or chronic disease) and post-intervention pain scores. This suggests that the beneficial effects of the combined intervention were consistent across participant subgroups, indicating generalizability of the treatment approach. Comparable findings have been reported by Nazari et al. (30), who found that the therapeutic effects of conservative physiotherapy modalities are not significantly influenced by demographic factors but are instead determined by the appropriateness and consistency of the applied technique. Emerging evidence from fascia-oriented rehabilitation research further supports these observations. Wolska et al. highlighted that self-myofascial release approaches, including foam rolling and tissue-focused interventions, may improve mobility, circulation, tissue flexibility, and functional recovery while reducing pain and muscle stiffness (26). Similarly, Klich et al. demonstrated that foam rolling and tissue flossing significantly reduced tendon stiffness and enhanced physical performance outcomes, likely through improved tissue extensibility and neuromuscular function (41). These physiological mechanisms may partly explain the enhanced functional improvements observed in the experimental group receiving cryotherapy combined with the Graston Technique in the present study.

These findings have important clinical implications. The integration of cryotherapy with IASTM can be considered an effective, non-invasive rehabilitation protocol for patients experiencing acute or subacute knee pain. By addressing both inflammatory and mechanical restrictions, this approach can improve functional independence and overall quality of life. Furthermore, the method is cost-effective, easy to administer, and safe when performed under professional supervision. However, the current study’s relatively small sample size and short duration limit generalization. Future studies with larger, randomized controlled designs and longer follow-up periods are warranted to confirm these effects and evaluate long-term functional outcomes. Additional research could also explore different cryotherapy durations, frequencies, and temperature ranges to optimize treatment efficacy.

A key strength of this study lies in its experimental design, which allowed for a direct comparison between cryotherapy combined with the Graston Technique and the Gras-ton Technique alone. The use of standardized pain and functional assessment tools, namely the VAS and ADL performance scores, ensured objective measurement of clinical outcomes. The study also maintained balanced demographic characteristics between groups, reducing confounding bias. Additionally, by focusing on short-term rehabilitation outcomes, the study highlights the immediate clinical utility of integrating cryotherapy into manual therapy practice for musculoskeletal knee pain.

### 4.1 Limitations

This study has several limitations. The study involved a relatively small sample size and short intervention period, limiting the ability to assess long-term outcomes. Participant blinding was not feasible because the cold sensation associated with cryotherapy could be recognized during treatment, which may have introduced expectation bias. Additionally, no objective physiological measures such as tissue temperature, muscle stiffness, or inflammatory biomarkers were assessed to directly confirm the proposed mechanisms of action. Future studies should incorporate larger randomized controlled trials, longer follow-up periods, and objective biomechanical or physiological measurements to further validate the effectiveness of combined cryotherapy and IASTM interventions.

### 4.2 Clinical implications

The results of this study have practical implications for rehabilitation professionals. Incorporating cryotherapy immediately after IASTM sessions can optimize recovery by minimizing post-treatment soreness, reducing inflammation, and improving range of motion. This combined approach can be particularly beneficial in early rehabilitation phases for patients with acute or subacute knee pain, allowing faster return to normal activities. Moreover, the protocol is non-invasive, low-cost, and easily applicable in clinical settings, making it suitable for integration into standard physiotherapy programs. Continued research in diverse populations such as athletes, older adults, and individuals with chronic pains recommended establishing standardized treatment parameters and validating the long-term benefits of this combined intervention.

## 5 Conclusion

This study demonstrates that combining cryotherapy with the Graston Technique can effectively reduce pain and improve functional recovery in individuals with knee joint pain compared with the Graston Technique alone. The combined approach appears to provide complementary therapeutic effects by reducing inflammation, improving soft-tissue mobility, and enhancing the ability to perform daily activities. As a non-invasive and relatively low-cost intervention, this treatment strategy may be valuable in both physiotherapy and rehabilitation nursing practice.

Future rehabilitation approaches may benefit from integrating AI-based technologies and digital health tools, such as motion-tracking systems, wearable feedback devices, and machine-learning–based recovery monitoring. These technologies could support more personalized rehabilitation by helping clinicians monitor patient progress, adjust treatment intensity, and predict recovery outcomes more effectively. In addition, further comparative studies involving other rehabilitation modalities, including heat therapy and neuromuscular stimulation, are needed to establish standardized and evidence-based treatment protocols that optimize patient recovery and long-term functional independence.

## Data Availability

The raw data supporting the conclusions of this article will be made available by the authors, without undue reservation.
